# Characterizing the effects of missing data and evaluating imputation methods for chemical prioritization applications using ToxPi

**DOI:** 10.1186/s13040-018-0169-5

**Published:** 2018-06-13

**Authors:** Kimberly T. To, Rebecca C. Fry, David M. Reif

**Affiliations:** 1 Bioinformatics Research Center, North Carolina State University, 1 Lampe Dr, Raleigh, 27695 NC USA; 2 Department of Environmental Sciences and Engineering, University of North Carolina at Chapel Hilll, Chapel Hill, 27516 NC USA; 3 Center for Human Health and the Environment, North Carolina State University, Raleigh, 27695 NC USA; 4 Department of Biological Sciences, North Carolina State University, Raleigh, 27695 NC USA

**Keywords:** Chemical prioritization, ToxPi, ToxCast, Missing data, Imputation, Multiple imputation, Simulation

## Abstract

**Background:**

The Toxicological Priority Index (ToxPi) is a method for prioritization and profiling of chemicals that integrates data from diverse sources. However, individual data sources (“assays”), such as in vitro bioassays or in vivo study endpoints, often feature sections of missing data, wherein subsets of chemicals have not been tested in all assays. In order to investigate the effects of missing data and recommend solutions, we designed simulation studies around high-throughput screening data generated by the ToxCast and Tox21 programs on chemicals highlighted by the Agency for Toxic Substances and Disease Registry’s (ATSDR) Substance Priority List (SPL), which helps prioritize environmental research and remediation resources.

**Results:**

Our simulations explored a wide range of scenarios concerning data (0-80% assay data missing per chemical), modeling (ToxPi models containing from 160-700 different assays), and imputation method (k-Nearest-Neighbor, Max, Mean, Min, Binomial, Local Least Squares, and Singular Value Decomposition). We find that most imputation methods result in significant changes to ToxPi score, except for datasets with a small number of assays. If we consider rank change conditional on these significant changes to ToxPi score, we find that ranks of chemicals in the minimum value imputation, SVD imputation, and kNN imputation sets are more sensitive to the score changes.

**Conclusions:**

We found that the choice of imputation strategy exerted significant influence over both scores and associated ranks, and the most sensitive scenarios were those involving fewer assays plus higher proportions of missing data. By characterizing the effects of missing data and the relative benefit of imputation approaches across real-world data scenarios, we can augment confidence in the robustness of decisions regarding the health and ecological effects of environmental chemicals

## Background

Current estimates of the number of chemical entities in commerce and the environment range from 7690 to 85000 [1, 2]. The enormous number presents challenges for a scientific and regulatory community tasked with assessing human and ecological health risks for each chemical. Given realistic limitations on time and resources for testing, methods for prioritizing and profiling the risk-relevant activity (both observed and predicted) of chemicals are needed for diverse application areas.

A prominent example of the need for resource prioritization is the Substance Priority List (SPL) generated in support of the U.S. EPA Superfund Program [3]. In 1980, the United States Congress enacted the Comprehensive, Environmental Response, Compensation and Liability Act (Superfund) in an effort to identify and clean hazardous waste dump sites and prevent future exposures to the general public. The Agency for Toxic Substances and Disease Registry (ATSDR) was thus established to “effectuate and implement the health related authorities” of the Superfund Act. The Superfund Amendments and Reauthorization Act of 1986 requires that ATSDR release a list of chemicals commonly found at Superfund sites listed on the National Priorities list, prioritized for further study.

The SPL is released every two years and prioritizes chemicals based on frequency at Superfund sites, toxicity, and potential for human exposure. Toxicity is quantified by drawing upon multiple, existing databases of hazardous substances. Provisions for expanding the source data include calls for inclusion of data from the U.S. EPA Toxicity Forecaster (ToxCast) and joint federal Tox21 programs [4]. The goal is to provide maximal information for prioritization of chemicals in the SPL.

Together, these programs conduct high throughput screening assays of thousands of chemicals. The first phase of ToxCast tested over 300 chemicals with extensive literature regarding toxicity. The second phase of ToxCast tested over 2000 chemicals in over 700 high throughput assays covering expansive pathways and endpoints. Subsequent phases are expanding into less well-studied chemical space. The data have been adopted to improve the profiling and prioritization of chemicals as a part of the EPA Endocrine Disruptor Screening Program (EDSP) and assessments by the World Health Organization’s International Agency for Cancer Research (IARC) [5–7].

However, even with the express goal of improving coverage, multisource data for any one compound will commonly involve “missing” data. This missingness can arise from inconclusive assay results, lack of testing in a particular assay, or incompatibility with a given assay system. In the aggregated matrix of all chemicals by all data (i.e. assays), the resulting missing data patterns can be complex. Moreover, these patterns may bias prioritization efforts, as chemicals having more complete information will tend to have inflated priority, shifting less well-studied chemicals to artificially lower ranks.

Data imputation procedures attempt to address missingness by using the structure of the global dataset to make informed estimates of values to replace those deemed missing. Imputation procedures are common in fields such as genetics, where the use of genotype imputation has been shown to increase the power of genome-wide association study (GWAS) scans by using known genotyped data to impute unsequenced single nucleotide polymorphisms (SNPs) for unrelated individuals [8]. In high-dimensional toxicological datasets, imputation may be used in a similar manner across collections of assays and chemicals. However, imputation methods for integrated data face special challenges, such as non-common data unique to each study, minimal common data across studies, or lack of common data [9]. In the context of the ToxCast data, this relates to differing assay cell types, assay targets, and chemicals assessed. Other groups have explored the effects of changes in integrated data on downstream analyses for multi-omics data, highlighting the benefits of using the relationships within multi-omic data for imputation [10].

Here, we explore the effects of complex missing data patterns on chemical prioritization using the Toxicological Prioritization Index (ToxPi) [11]. We then implement several data imputation methods and characterize their performance across a range of scenarios. We develop and test our methods using simulated data motivated by high-throughput screening (HTS) data generated on chemicals in the SPL. We use 7 imputation methods on data generated using 1000 simulations each across 36 scenarios, then compare results back to the original data. Our results show that, even in the absence of explicit choices to impute missing data, ignoring missingness may still exert effects on prioritization conclusions. Therefore, knowledge of the effects of missingness and imputation across scenarios will be essential to providing robust prioritization and profiling decisions.

## Methods

### Data

ToxCast data were obtained from the October 2015 EPA high throughput screening data release [12]. Chemicals featured on the 2011 ATSDR Substance Priority List were obtained from the ATSDR website [3]. Chemicals from the ToxCast negative log transformed AC50 value matrix were then compared to those chemicals on the Substance Priority List, yielding 426 shared chemicals. Assays with 100% missing data resulting from the removal of non-overlapping chemicals were removed from further analysis. This yields a final working dataset of negative log transformed AC50 values for 426 chemicals from 1092 assays. The 1092 assays were from different source technology platforms, the details of which are available in the ToxCast Data Supporting Documents [12]. The range of missing data for the 426 chemicals is (0%, 94.14%) and for the 1092 assays is (3.76%, 97.18%). Use of the negative log transformed AC50 values guarantees that the source of missing data is a lack of testing from the assay source group as this data matrix accounts for inactive hits numerically. For the purpose of the simulations, chemicals and assays were given arbitrary identifiers.

### Data simulation

**Fig. 1:**
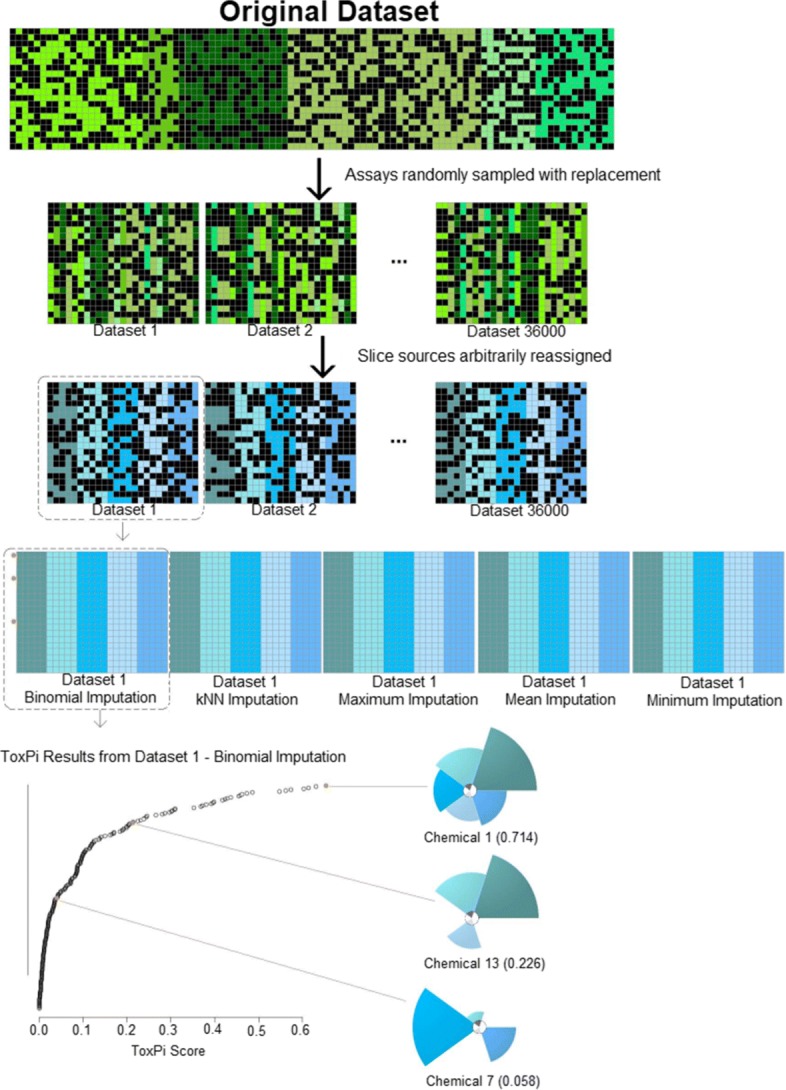
Conceptual overview of the simulation process and experimental design. Assays were randomly sampled from the original data based on a desired number of assays and assay sources (slices) so that the simulated datasets contained a subset of assays with arbitrarily assigned sources and all of 426 chemicals present in the original dataset. Simulated datasets were imputed and ToxPi profiles were calculated, with an overall summed ToxPi score given for analysis

Prior to removing the assay names, assay counts were recorded to determine parameters for the data simulation. The working dataset features 12 unique assay sources, with a range of (1420) assays per source. Within the ToxPi framework, each assay source represents ToxPi “Slices” and each assay would be included in scoring within the assay source. For the simulation we used *s*=(5,10,15,20) assay sources, deemed “Slices.” Within each assay source, the simulation includes *a*=(1,5,10,15,20,30,50,90,125) assays. Figure 1 illustrates a ToxPi model for *s*=5 and *a*=5, where the individual assays (single columns within the data matrix) were recombined into summary source-wise scores (slices). Assays were randomly sampled and assigned an assay source to yield simulated datasets of size 426 × (s*a). Each combination of s*a was repeated 1000 times, yielding 36,000 simulated datasets.

### Data imputation

Data were imputed using assay values to maintain the assay screening patterns across chemicals. *Mean imputation* imputes the individual assay means. *Minimum value imputation* imputes the minimum assay readout, which assumes that chemicals with missing data within an assay were at most, minimally active. *Maximum value imputation* imputes the maximum assay readout, which assumes that the chemicals with missing values were the most active within each assay. *kNN imputation* uses the assay means from the *k* most highly correlated chemical for each individual missing data point. The value of *k* is calculated as the ceiling of $\sqrt {number \ of \ assays}$, as recommended by Duda, Hart, and Stork [13]. In instances where there were fewer than *k* correlated assays, chemicals were considered inactive and imputed with 0. Correlation between chemicals was calculated using Pearson’s Correlation Coefficient on pairwise complete observations in the working dataset. *Binomial imputation* converts the continuous AC50 values to a dichotomous response rate where inactive reads remain 0 and any reads greater than 0 are converted to 1. The proportion response rate is calculated and missing values are imputed as inactive or active (0 or 1, respectively) using a binomial probability with the calculated proportion response rate. *Local Least Squares imputation* (LLS) selects the *k* most highly correlated assays and performs a local least squares minimization to predict missing assay values. A single value, *k*=2 was chosen to demonstrate the effects of LLS on chemical ranking and scores. The *k* most similar assays were selected by Pearson Correlation Coefficient. LLS was initiated by imputing assay means and iterating the local least squares calculation until the difference in iterated datasets, as calculated by mean square error, is below the threshold value 0.001. *Singular Value Decomposition imputation* (SVD) predicts assay values by performing matrix decomposition on the original dataset and recombines the results into a solution. SVD imputation is initiated by imputing 0 and iterates through singular value decompositions of the updated datasets until convergence. Imputation on the original working dataset used the ToxPi default of ignoring missing data by imputing 0 for all missing data. In the chemical ranking scheme, this default procedure essentially assumes inactivity where there exists missing data. All seven imputation methods were implemented on the 36,000 simulated datasets, yielding 252,000 simulated complete datasets for 252 unique assay-slice-method combinations.

### ToxPi calculation

Chemicals within each simulated dataset were scored and ranked using the standard ToxPi formula, wherein half maximal active concentrations (AC50) for assays with common targets are summed for each individual chemical. Total summations for each chemical are normalized to the interval (0, 1) [14]. Each assay and slice was weighted equally for all combinations of assay- and slice-numbers simulated. Higher ToxPi scores are given to those chemicals deemed more active (slices extending farther from the origin in Fig. 1), and lower ToxPi scores are given to those chemicals deemed least active (slices not extending far from the origin in Fig. 1) or inactive (slices having zero radius, in Fig. 1). ToxPi Scores are sorted and ranked so that the chemical with the highest ToxPi score has rank 1. ToxPi was also run on the imputed working dataset to serve as a baseline for evaluating imputation effects on ToxPi results.

### ToxPi score and rank evaluation

Root mean square error (RMSE) was used to compare ToxPi scores of the imputed simulated datasets with ToxPi scores of the imputed working dataset. Higher RMSE indicates larger differences between datasets imputed with the test methods versus the standard ToxPi imputation method. A narrower range of RMSE values for each slice-assay-imputation combination indicates more stability in imputation method. Likewise, a wider range of RMSE values for each combination indicates less stability and therefore reliability in imputation method.

$$RMSE = \sqrt{\frac{\sum_{i=1}^{m}{(y_{i}-y_{0})^{2}}}{m}} \,$$

$$where \ m \ = \ number\ of \ chemicals $$

$$y_{ij} \ = \ ToxPi \ Score \ of \ Chemical \ i \ using \ imputation \ method \ j \, $$

$$y_{i0} \ = \ ToxPi \ Score \ of \ Chemical \ i \ using \ imputed \ working \ dataset \ $$

Additionally, ToxPi scores of the imputed simulated datasets were compared with ToxPi scores of the imputed working dataset using the Wilcoxon Signed-Ranks Test for paired observations, which considers the distribution of differences in paired ToxPi scores. Mean ToxPi Scores were calculated for each unique assay-score-method combination group of 1,000 simulated datasets for each of 426 chemicals, yielding 252 ToxPi score means. P-values from the Wilcoxon Signed-Ranks test were evaulated against Bonferroni corrected significance levels such that *α*=0.001/252. *P*-values less than *α* indicate that the paired ToxPi Scores from the imputed simulated datasets and from the imputed working dataset are significantly different.

The variance of ToxPi scores was calculated for each individual chemical among datasets with the same slice number, assay number, and imputation methods so that each scenario resulted in 426 different variance values. Larger variation indicates less score stability as a result of the combination of parameters.

Overall ToxPi score variance across all chemicals for each scenario was tested against the variance of ToxPi scores from the raw imputed data using Bartlett’s test for equal variances. Resulting *p*-values were tested against the Bonferroni corrected significance level *α*=0.001/252,000.

ToxPi ranks for the imputed simulated datasets were compared with ranks from the original ToxPi results. Larger values in rank change indicate greater differences in rank. Likewise, smaller values indicate smaller to no difference in rank from the choice of imputation method.

$$Mean \ Rank \ Change = (r_{ij} - r_{i0})/1000 $$

$$where \ r_{ij} \ = \ Rank \ of \ Chemical \ i \ using \ imputation \ scenario \ j; $$

$$r_{i0} \ = \ Rank \ of \ Chemical \ i \ from \ imputed \ working \ dataset $$

### Statistical software

Scripts for generating simulated datasets, data imputation, running ToxPi and statistical analyses were used, written and run using R Statistical Software [15]. Graphs were made using the ggplot2 package in R.

## Results

### Missing data

In the original dataset, the range for missing data amongst chemicals is (0%, 94.14%). Across assays, the range for missing data is (3.756%, 97.18%).

### Wilcoxon signed-rank tests show most imputation methods result in significant changes to score

The Wilcoxon Signed-Rank test was significant at the Bonferroni corrected *α*=0.001/252 for all tests except in cases with smaller simulated datasets. Minimum value imputation scores appear to be more sensitive in cases were there are a fewer number of assays (i.e. when number of assays per slice = 1), whereas mean and kNN imputation only showed nonsignificance in the smallest dataset (5 slices, 1 assay per slice). However, the remaining methods showed no such pattern, and the results of the Wilcoxon Signed-Rank test indicate much greater sensitivity to the randomness of the simulation.

### Minimum value imputation serves as a baseline for comparison

In the original dataset, each assay has the same minimum value of 0. Therefore, the ToxPi default and minimum value imputation are identical when using the same dataset. Because simulated datasets are generated from a diverse number of randomly sampled assays from the original dataset, variability is expected between chemical ToxPi scores from the minimium value imputed simulated datasets and scores from the standardly imputed original dataset. Besides scenarios where the number of assays per slice is 1, every other result shows scores significantly different than those from the ToxPi default (*p*<0.001/252). The results from the minimum value imputation can thus be used as a baseline for comparing methods.

**Fig. 2:**
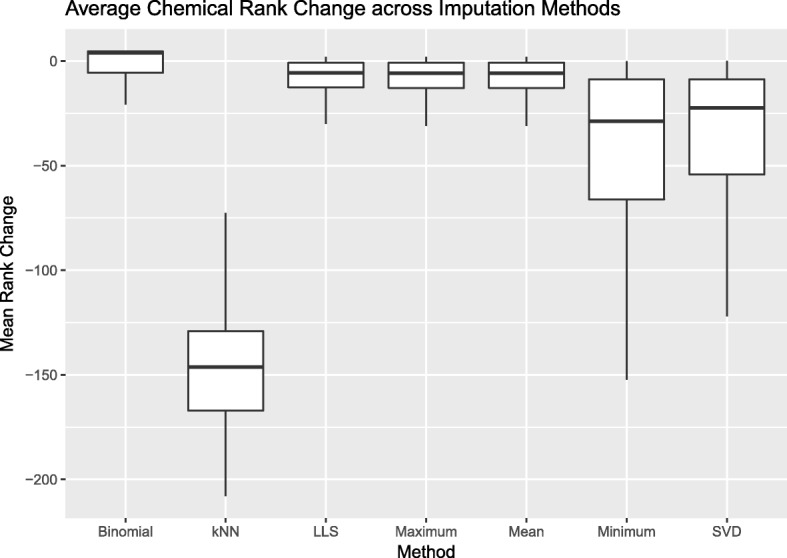
Comparison of Imputation Methods Using ToxPi priority ranks. Mean ToxPi Rank Change between Imputed Simulated Data and Imputed Raw Data. Rank change was calculated by using the magnitude of difference between individual chemical ranks in the imputed raw data and the chemical ranks from each simulated dataset. Binomial, LLS, Maximum, and Mean show small magnitudes of change in rank. kNN shows a wider variation in rank change and therefore represents less stability in the method. Minimum value imputation and SVD present wider ranges in rank change, although the magnitude of change is smaller than kNN

**Fig. 3:**
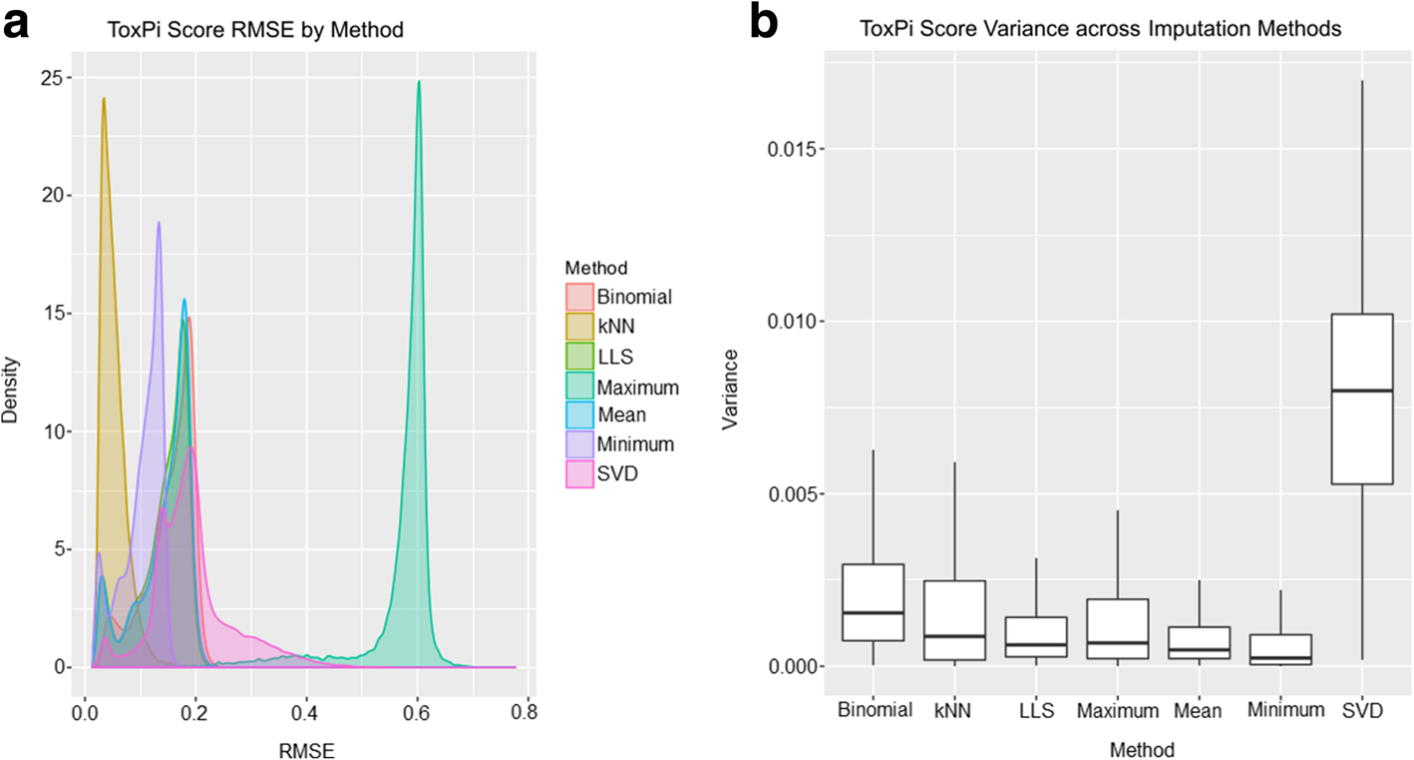
Comparison of Imputation Methods by Toxpi Score. **a** Root Mean Square Error between Imputed Simulated Data ToxPi Scores and Imputed Raw Data Chemical Scores. After imputation and ToxPi calculation, scores were compared to the ToxPi scores using the standard “0” method. RMSE density distributions are separated by imputation method. The distribution of kNN is centered at the lowest RMSE compared to the other methods. Binomial, LLS, and Mean imputation are heavily overlapped. SVD is centered similarly, but shows a wider spread. Maximum imputation has the largest RMSE. **b** ToxPi Score Variance of Imputed Simulated Data ToxPi Scores. Amongst 1000 replicate simulations, the variance for each of 426 chemicals was calculated. Compared to SVD, all other methods present relatively low variability from chemical to chemical. SVD has an extremely wide range of ToxPi Score variability

Minimum value imputation shows relatively small effects on chemical scores (Fig. 3a), with low variability in RMSE across imputations (Fig. 3b). However, these small shifts in scores have a highly variable effect on chemical ranks (Fig. 2).

### kNN causes unstable shifts in chemical ranks

RMSE between ToxPi scores from kNN imputed datasets and the original dataset presented the smallest values compared to all other imputation methods (Fig. 3a). Despite these low changes in score, kNN imputation overall has the most drastic effect on rank change for each chemical (Fig. 2), highlighting the instability of this method for the purposes of ranking.

### Maximum value imputation causes uniform shifts in score and no shift in rank

Scores derived with maximum value imputed data differ largely with smaller number of assays per slice (Table 1). Overall, maximum value imputation demonstrates large differences from the original ToxPi imputed data (Fig. 3a), however the variance of these scores remains comparable to the most of the remaining methods. Although the difference between ToxPi scores is significant (*p*<0.001/252), changes in rank are lower in magnitude and more stable than minimum value imputation (Fig. 2). This indicates a uniform change in scores across chemicals, resulting in little changes in rank.

**Table 1:** Values for the range of RMSE and range of Rank Change for each of 7 imputation methods, separated by the number of assays per slice

Slices/Assay										
Method	Measure	1	5	10	15	20	30	50	90	125
Binomial	Rank Change	(-210,-40)	(-150,2)	(-74,4.2)	(-37,4.5)	(-26,4.6)	(-15,4.6)	(-8.8,4.6)	(3.2,4.6)	(3.1,4.6)
Binomial	RMSE	(0.024,0.35)	(0.058,0.25)	(0.083,0.27)	(0.086,0.24)	(0.12,0.26)	(0.12,0.25)	(0.13,0.24)	(0.15,0.24)	(0.16,0.24)
kNN	Rank Change	(-210,-57)	(-210,-59)	(-210,-55)	(-210,-55)	(-210,-62)	(-210,-61)	(-210,-57)	(-210,-48)	(-210,-45)
kNN	RMSE	(0.016,0.16)	(0.017,0.23)	(0.017,0.22)	(0.019,0.2)	(0.018,0.22)	(0.018,0.25)	(0.019,0.22)	(0.019,0.21)	(0.017,0.2)
LLS	Rank Change	(-190,-12)	(-56,-1.7)	(-27,-0.31)	(-25,1)	(-19,1.4)	(-14,1.6)	(-11,2)	(-8.7,2)	(-6.6,2)
LLS	RMSE	(0.019,0.23)	(0.043,0.43)	(0.077,0.41)	(0.09,0.45)	(0.054,0.42)	(0.1,0.45)	(0.13,0.31)	(0.14,0.24)	(0.14,0.23)
Maximum	Rank Change	(-54,-1.7) (-210,-11)	(-29,0.53)	(-22,0.88)	(-18,1.4)	(-14,1.8)		(-11,2)	(-8.3,2)	(-6.4,2)
Maximum	RMSE	(0.031,0.78)	(0.36,0.76)	(0.45,0.68)	(0.46,0.67)	(0.5,0.66)	(0.54,0.65)	(0.55,0.64)	(0.57,0.63)	(0.57,0.64)
Mean	Rank Change	(-210,-11)	(-54,-1.7)	(-29,0.53)	(-22,0.88)	(-18,1.4)	(-14,1.8)	(-11,2)	(-8.3,2)	(-6.4,2)
Mean	RMSE	(0.019,0.26)	(0.043,0.23)	(0.069,0.28)	(0.08,0.24)	(0.097,0.26)	(0.1,0.24)	(0.12,0.24)	(0.14,0.23)	(0.15,0.23)
Minimum	Rank Change	(-210,-57)	(-180,-21)	(-25,0)	(-110,-7.5)	(-91,-4.8)	(-73,-1.9)	(-50,-0.76)	(-31,0)	(-140,-9.2)
Minimum	RMSE	(0.014,0.14)	(0.031,0.16)	(0.047,0.15)	(0.065,0.16)	(0.074,0.16)	(0.086,0.17)	(0.093,0.17)	(0.11,0.17)	(0.11,0.17)
SVD	Rank Change	(-210,-49)	(-160,-17)	(-130,-5.5)	(-97,-4.4)	(-81,-3.4)	(-64,-1.2)	(-51,-0.15)	(-34,0.15)	(-25,0.15)
SVD	RMSE	(0.02,0.46)	(0.04,0.62)	(0.073,0.54)	(0.097,0.54)	(0.096,0.57)	(0.11,0.54)	(0.1,0.54)	(0.12,0.53)	(0.11,0.53)

### Mean, binomial, and LLS imputation show similar effects

The RMSE distributions for Mean, Binomial, and LLS imputation show highly overlapping distributions (Fig. 3a). Similar to maximum value imputation, Mean, Binomial, and LLS imputation have relatively stable effects on ToxPi score (Fig. 3b), with overall significant differences from the original ToxPi imputed dataset (*p*<0.001/252). Similar to maximum value imputation, these changes in ToxPi score had only small effects on average chemical rank change (Fig. 2).

### Variance of scores is stable in all methods except SVD

Variance of individual chemical ToxPi scores across datasets and imputation methods are plotted in Fig. 3b. Overall, the variance of ToxPi scores is low for all imputation methods (<0.02), however, the results from SVD appear highly variable. The center of the distribution for the RMSE of SVD imputed datasets is similar to that of Mean, Binomial, and LLS (Fig. 3a), however, the spread of this distribution is much wider (Fig. 3b). The wide range of RMSE may indicate instability in this method. Because SVD imputation begins with mean imputation, there may be effects of the data structure that alter factorization of data matrices from dataset to dataset. Further, the average chemical rank change has higher variability than other imputation methods (Fig. 2).

### Overall ToxPi score variance differs significantly

Results from Bartlett’s Test showed that all datasets from Binomial, Maximum, and Minimum imputation have significantly different variances amongst ToxPi scores when compared to the original raw data results. LLS, Mean, and SVD each had >90% of datasets with significantly different variances amongst ToxPi scores. KNN Imputation had 85% of datasets with significantly different variances. These results show that there are observed variance differences between the ToxPi scores from the imputed datasets compared to the default ToxPi imputation method.

## Discussion

Improvements to the handling of missing data in chemical prioritization cases will improve the selection of chemicals for further study and ultimately decrease the time and resources needed to characterize these chemicals on a case-by-case basis. The optimal imputation method will feature score stability across all possible datasets, low sensitivity to smaller adjustments as a result of the imputation, and an ability to yield accurate ranking of chemicals. Because this simulation seeks to improve chemical ranks that will lead to further testing of chemicals deemed highest priority, we are unable to definitively determine if the resulting rankings are accurate. However, by improving the imputation method based on the first two criteria, we can begin to extract information that is available in the non-missing toxicity measurements that will improve the scoring and relative rankings.

With this original dataset, minimum value imputation behaves identically to the ToxPi default. However, this is not a guarantee for all datasets, where the minimum value may differ across assays. In this simulation, the variance of scores from minimum value imputation is low (Fig. 3b). Likewise, the RMSE is low, indicating small differences between ToxPi scores from simulated datsets and scores from the original dataset (Fig. 3a). However, the Wilcoxon Signed Rank Test for paired observations indicates significant difference between scores, except for cases with 1 assay per slice. Rank changes from minimum value imputation show greater variation than all other imputation methods (Fig. 2), indicating that minimum value imputation is sensitive to small changes in score.

The kNN imputation method resulted in the smallest changes in ToxPi score, albeit a wider ranger in score variability compared to Binomial, LLS, Maximum, Mean, and Minimum. kNN imputation also had the highest magnitude of rank changes despite the minimal changes to ToxPi score, indicating unwanted sensitivity to small changes in score. This may be due to the method’s dependence on correlations, which cannot be measured on assays with no variation and therefore the possibility of excluding important data is present. In this simulation, we tested a single value for *k* (*k*=10). It is possible that larger values of *k* may extract more information from the data for imputation, however the missingness structure of the original data limits the selection of a greater number of neighbors.

Maximum value imputation showed low variance among scores, large changes in scores, but almost no change in ranks. This indicates that the uniform changes in score cause a global increase in ToxPi scores leading to little or no effect on rank. Therefore, this method can not be recommended over the current ToxPi default.

Binomial, LLS, and Mean imputation performed similarly to maximum value imputation, showing lower variance among scores, stable changes in scores, and almost no change in ranks to the current method. Additionally, compared to minimum value imputation, binomial, LLS, and mean imputation have higher RMSEs overall and slightly wider ranges of RMSE. The lower RMSE and narrower range of RMSE for minimum value imputation indicates that there is no new information being extracted via minimum imputation. The greater RMSEs and wider ranges of RMSEs can be considered reasonably different, relative to the results of the remaining imputation methods. That is, the differences achieved using these methods justify recommendations for improvements to the ToxPi default imputation method.

SVD imputation showed unique results, with higher variance in individual chemical ToxPi scores across scenarios. Despite this higher variability, we see a narrower range of rank changes compared to minimum value imputation indicating that SVD imputation results in highly variable score distributions with moderate sensitivity to chemical ranks. Because SVD decomposes the data, it is possible that this imputation method is able to extract additional data that yields these unique results. Further exploration into the accuracy of SVD imputation for the purposes of ToxPi chemical scoring and ranking is needed.

Based on the results of this simulation, recommendations for binomial imputation versus LLS imputation or mean imputation are arbitrary because of the similar RMSEs and minimal changes in rank. Results from kNN imputation demonstrate that the imputed values were selected from the same *k*=10 nearest neighbors for each chemical, resulting in ties for many chemicals that would explain the larger magnitude of rank change. Future work may explore different values of k for more stable adjustments in rank. Similarly, we chose to explore a single value of *k* for LLS. Higher values for *k* have been shown to produce different results [16], and may differentiate LLS results from Mean or Binomial imputation.

## Conclusions

In conclusion, we recommend that in cases where chemical rank is of greatest concern, mean imputation be used in place of the current ToxPi standard of ignoring missing data. Mean imputation shifts each chemical’s ToxPi score centrally, allowing for the more stringent assumption of some constant activity. Currently, imputing “0” for all missing data relies on the assumption that a chemical is always in its minimally active state, which may be too lenient when considering human and environmental health with risk assessment. It is shown that mean imputation, LLS imputation, and binomial imputation behave similarly in regards to score, however the effects on rank are dependent on the number of slices and assays. Therefore, binomial imputation is a comparable alternative to mean imputation when chemical ranking is not to be considered. Future studies might explore external chemical meta-data to impute results from more structurally similar chemicals or look to more model-based Bayesian approaches to utilize distributional knowledge from particular assay sets.

## Abbreviations

ATSDR: Agency for toxic substances and disease registry
GWAS: Genome-wide association study
HTS: High-throughput screening
kNN: k-nearest neighbors
LLS: Local least squares
RMSE: Root mean square error
SNP: Single nucleotide polymorphism
SPL: Substance priority list
SVD: Singular value decomposition
ToxCast: Toxicity forecaster
ToxPi: Toxicological priority index
